# Cohesin-mediated loop extrusion and enhancer-associated factors additively contribute to *Sox2* looping with its distal enhancer

**DOI:** 10.1101/gad.353296.125

**Published:** 2026-06-01

**Authors:** Moreno Martinovic, Koen D. Flach, Marit A.C. de Kort, Michela Maresca, Hans Teunissen, Ning Qing Liu, Tineke L. Lenstra, Elzo de Wit

**Affiliations:** 1 Division of Gene Regulation, The Netherlands Cancer Institute, Amsterdam 1066 CX, The Netherlands;; 2 Oncode Institute, Utrecht 3521 AL, The Netherlands;; 3 Department of Clinical Genetics, Erasmus University Medical Center, Rotterdam 3015 GD, The Netherlands;; 4 Department of Hematology, Erasmus Medical Center Cancer Institute, Rotterdam 3015 GD, The Netherlands

**Keywords:** 3D genome, enhancers, genomics, loop extrusion, pluripotency

## Abstract

*Sox2* expression is regulated by a distal enhancer cluster known as the *Sox2* control region (SCR). Although *Sox2* and the SCR exhibit strong interactions in chromosome conformation capture experiments, acute depletion of cohesin only mildly affects *Sox2* transcription. Here we show that continuous restarting of loop extrusion, but not stalling of cohesin at CTCF sites, is involved in the maintenance of the *Sox2*–SCR loop. While depletion of cohesin or CTCF has only a mild impact on *Sox2* gene expression, stabilizing cohesin on the DNA by depleting the cohesin release factor WAPL results in a rapid decrease in expression. Furthermore, using genome editing, we show that a weak enhancer located downstream from the *Sox2* gene renders *Sox2* expression insensitive to the loss of cohesin stalling at CTCF sites. Finally, through targeted and pleiotropic inhibition of the SCR, we demonstrate that the *Sox2*–SCR loop is a multicomponent system that depends on cohesin-mediated loop extrusion and enhancer-associated factors. Together, our findings highlight the intricate interplay between enhancer activity and loop extrusion in the establishment and maintenance of chromatin loops in the *Sox2* locus.

## Abstract (precis)

In this study, Martinovic et al. provide mechanistic insight into the enhancer–promoter interactions at the Sox2 gene locus. Continuous loop extrusion through dynamic cohesin turnover and BET protein binding to the Sox2 enhancer cluster SCR independently and additively contribute to the Sox2–SCR looping that is required for Sox2 expression.

Gene regulation in multicellular organisms is orchestrated by a complex interplay of promoters and distal regulatory elements, particularly enhancers. Enhancers are noncoding DNA elements that ensure correct spatial and temporal expression of many genes during development and in adult tissues (Shlyueva et al. 2014). Enhancers can activate expression of genes over genomic distances of hundreds of kilobases (Schoenfelder and Fraser 2019), such as in the *Shh* and *Pitx1* loci in limb development (Lettice et al. 2003; Kragesteen et al. 2018) or in *Sox9* in the developing gonad (Gonen et al. 2018). While many developmentally regulated genes are controlled by distal regulatory elements (Wu et al. 2021), how enhancers activate transcription over such large distances is still incompletely understood.

To activate transcription, enhancers must communicate with their target promoters. This long-range communication is thought to involve the 3D folding of the genome, bringing distal elements into proximity through the formation of chromatin loops (Rao et al. 2014). One of the key mechanisms driving chromatin loop formation is cohesin-mediated loop extrusion, in which cohesin complexes act as DNA motors to progressively increase loop size (Sanborn et al. 2015; Fudenberg et al. 2016; Davidson et al. 2019; Kim et al. 2019). In vertebrates, loop extrusion is typically halted when cohesin encounters CTCF bound to DNA at sites arranged in a convergent orientation (Guo et al. 2012; de Wit et al. 2015; Sanborn et al. 2015; Li et al. 2020; Nora et al. 2020). This process is thought to bring distal enhancers into proximity with their target genes to activate the target gene transcription, as evidenced by studies showing that cohesin is required for activation of genes by distal enhancers (Kane et al. 2022; Rinzema et al. 2022).

However, recent studies challenge this view, suggesting that enhancer–promoter interactions may largely persist in the absence of cohesin, raising questions about the general requirement of loop extrusion for distal gene regulation (Aljahani et al. 2022; Hsieh et al. 2022). These findings suggest that alternative mechanisms, such as phase separation (Hnisz et al. 2017; Boija et al. 2018; Sabari et al. 2018) or formation of microcompartments (Goel et al. 2023), may mediate enhancer–promoter communication independently of cohesin.

One context in which such mechanisms may play a key role is the regulation of pluripotency genes. In mouse embryonic stem cells (mESCs), pluripotency transcription factors such as SOX2, OCT4, and NANOG bind to clusters of enhancers associated with pluripotency-related genes, termed superenhancers (Hnisz et al. 2013; Whyte et al. 2013). These enhancer clusters have been proposed to create dynamic hubs of transcriptional activity that stabilize enhancer–promoter contacts and promote robust gene expression (Hnisz et al. 2017; Boija et al. 2018; Sabari et al. 2018).

SOX2 is a pioneer transcription factor that is crucial for the maintenance of the pluripotent state (Avilion et al. 2003; Boyer et al. 2005). The expression of *Sox2* in mESCs is strongly dependent on a distal downstream enhancer cluster, called the *Sox2* control region (SCR) (Li et al. 2014; Zhou et al. 2014; Taylor et al. 2022; Brosh et al. 2023). It has been previously shown that *Sox2* strongly interacts with the SCR in chromosome conformation capture experiments (de Wit et al. 2015; Aljahani et al. 2022; Taylor et al. 2022; Goel et al. 2023). The *Sox2* locus also contains several CTCF binding sites upstream of the *Sox2* gene and in the SCR, oriented in a convergent manner consistent with cohesin-mediated loop formation (Fig. 1A; de Wit et al. 2015). Interestingly, acute depletion of cohesin has been shown to have a mild impact on *Sox2* transcription (Aljahani et al. 2022) and to specifically impair transcriptional burst enhancement while leaving basal bursting unaffected (Du et al. 2024). Furthermore, live-cell imaging experiments failed to find a correlation between Sox2–SCR proximity and *Sox2* transcription dynamics (Alexander et al. 2019). These observations bring into question whether cohesin is essential for the regulatory function of the *Sox2*–SCR interaction.

**Figure 1.**
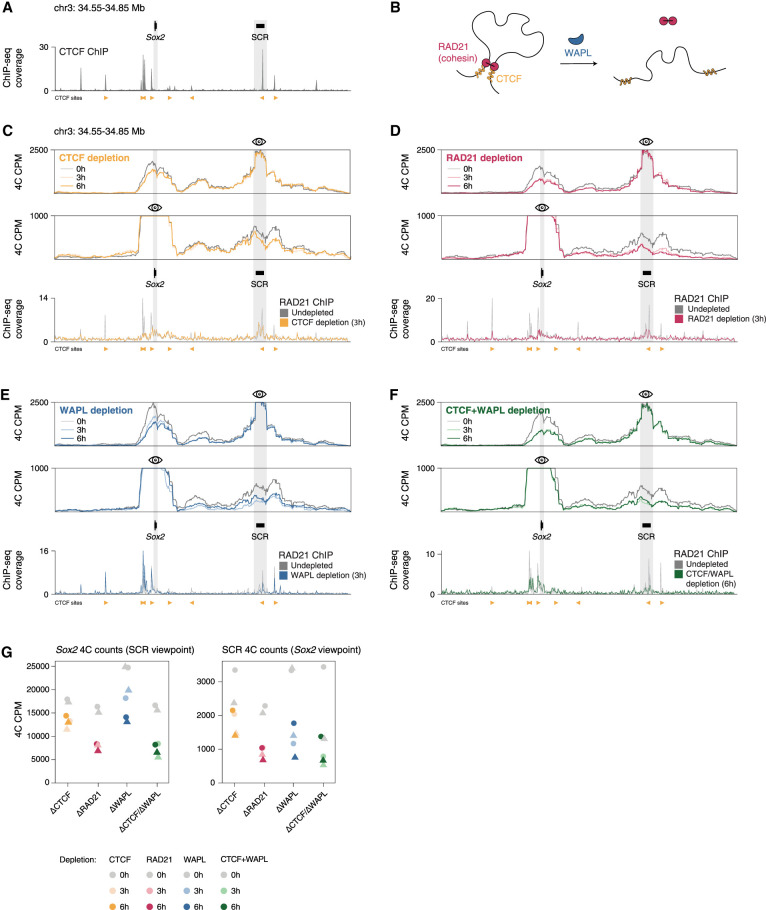
Continuous restarting of loop extrusion, but not stalling of cohesin at CTCF sites, maintains the *Sox2*–SCR loop. (*A*) Normalized CTCF ChIP-seq coverage is from Hsieh et al. (2022) in the *Sox2* locus, with bound CTCF sites shown *below*. The genomic locations of the *Sox2* gene and the SCR are indicated. (*B*) Schematic depiction of the 3D genome regulatory proteins that were acutely depleted, including cohesin, CTCF, and the cohesin release factor WAPL. (*C*) Normalized 4C-seq coverage using the SCR (*top*) and the *Sox2* promoter (*middle*) viewpoints, as well as RAD21 ChIP-seq coverage (*bottom*) in the Sox2 locus in the CTCF-AID cell line with and without CTCF depletion. ChIP-seq data are from Nora et al. (2020). (*D*) As in *C* but for the RAD21-AID cell line. ChIP-seq data are from Hsieh et al. (2022). (*E*) As in *C* and *D* but for the WAPL-AID cell line. ChIP-seq data are from Liu et al. (2021). (*F*) As in *C*–*E* but for the WAPL/CTCF-AID cell line. ChIP-seq data are from Liu et al. (2025). (*G*) Quantification of normalized 4C counts in individual replicates in the SCR using the *Sox2* viewpoint and in the *Sox2* gene using the SCR viewpoint across different depletions. Individual replicates are shown as points of different shapes.

In this study, we aimed to dissect how enhancer–promoter interactions are formed at the *Sox2* locus and what factors are required to ensure robust *Sox2* expression in mESCs. By leveraging acute protein depletion using degron systems, we found that CTCF and cohesin are largely dispensable for *Sox2* expression, while stabilization of cohesin on the DNA by removing the cohesin release factor WAPL—especially when combined with CTCF depletion—results in a rapid decrease in expression. Deletion of a weak enhancer downstream from the *Sox2* gene renders *Sox2* expression sensitive to the loss of cohesin stalling at CTCF sites. Through chemical inhibition of BET proteins and allele-specific epigenetic silencing of the SCR, we show that cohesin-mediated loop extrusion and active enhancer-associated factors additively contribute to *Sox2*–SCR looping. These findings show that disrupting enhancer–promoter looping through different mechanisms can yield distinct transcriptional outcomes and highlight the intricate interplay between loop extrusion and enhancer activity in the establishment and maintenance of chromatin loops and gene expression.

## Results

### Continuous restarting of loop extrusion, but not stalling of cohesin at CTCF sites, maintains the Sox2–SCR loop

To understand the role of different regulators of the 3D genome (Fig. 1B) in the interaction between *Sox2* and the SCR, we made use of the auxin-inducible degron (AID) system, in which the addition of IAA (auxin) induces rapid degradation of degron-tagged proteins (Nishimura et al. 2009). We used established mouse embryonic stem cell (mESC) AID lines for CTCF (Nora et al. 2017), the cohesin subunit RAD21 (Liu et al. 2021), the cohesin release factor WAPL (Liu et al. 2021), and a WAPL/CTCF double-degron line (van Schaik et al. 2022; Liu et al. 2025). We performed high-resolution in situ 4C experiments (van de Werken et al. 2012) from viewpoints at the *Sox2* promoter and the SCR in untreated cells and cells treated with IAA for 3 or 6 h. To our surprise, acute depletion of CTCF showed only a mild effect on the *Sox2*–SCR interaction (Fig. 1C,G). This was not due to incomplete degradation of CTCF, as GFP level was maximally decreased already after 3 h of CTCF depletion (Supplemental Fig. S1A). Furthermore, RAD21 chromatin immunoprecipitation followed by qPCR (ChIP-qPCR) showed that cohesin binding at a CTCF site downstream from the SCR was reduced upon CTCF depletion to a degree comparable with that of direct RAD21 depletion (Supplemental Fig. S1B,C). This is consistent with chromatin immunoprecipitation followed by sequencing (ChIP-seq) data in a CTCF-AID line (Hsieh et al. 2022) also displaying rapid resolution of RAD21 peaks outside of the SCR after 3 h of CTCF depletion (Fig. 1C, bottom). These results suggest that CTCF-mediated cohesin stalling is not the dominant factor in forming the *Sox2*–SCR interaction.

Previous work has shown that cohesin is required for the maintenance of the *Sox2*–SCR loop (Aljahani et al. 2022). To confirm that the cohesin complex plays a role in the formation of *Sox2*–SCR contacts in our system, we generated 4C profiles in a RAD21-AID line. Depletion of RAD21 for 3 h resulted in a reduction of the *Sox2*–SCR contact frequency to approximately half of the undepleted levels, while a depletion of 6 h did not lead to a further reduction (Fig. 1D,G).

We have previously shown that the stabilization of cohesin on chromatin by depleting WAPL results in the loss of cohesin from enhancers (Liu et al. 2021). To understand what happens to the *Sox2*–SCR interaction following WAPL depletion, we performed 4C experiments in WAPL-AID cells. We found that acute depletion of WAPL also resulted in a substantial decrease in the *Sox2*–SCR contact frequency (Fig. 1E,G). Therefore, both the loss of cohesin from chromatin and the stabilization of cohesin on chromatin result in a similar effect on the *Sox2*–SCR interaction. Although seemingly paradoxical, these results are consistent with our previous results that showed that WAPL depletion results in cohesin redistribution from enhancer regions to CTCF sites (Liu et al. 2021).

Redistribution of cohesin upon WAPL depletion was also reflected in increased cohesin binding at the CTCF sites upstream of *Sox2*, as shown by RAD21 ChIP-seq in WAPL-depleted cells (Fig. 1E, bottom; Liu et al. 2021). While the loss of CTCF in the presence of WAPL showed only a mild effect on the *Sox2*–SCR interaction, CTCF-mediated stalling of cohesin could still play a role in maintaining the remaining specific *Sox2*–SCR interactions upon WAPL depletion. To investigate this, we generated 4C profiles in a CTCF/WAPL-AID line (van Schaik et al. 2022; Liu et al. 2025). We found that combined depletion of CTCF and WAPL did not lead to a further reduction in *Sox2*–SCR contact frequency compared with WAPL depletion alone despite reduced cohesin accumulation at CTCF sites (Fig. 1F,G). Taken together, these results suggest that the specificity of the *Sox2*–SCR interaction is maintained in part by dynamic cohesin turnover but not by stalling of cohesin at CTCF sites.

### CTCF and cohesin, but not WAPL, are dispensable for the expression of Sox2

**Figure 2.**
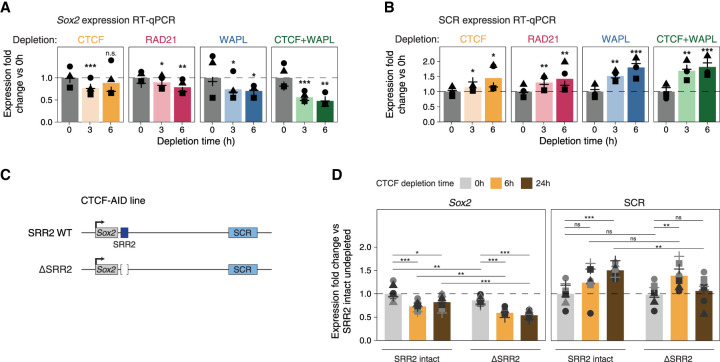
Different modes of *Sox2*–SCR loop perturbation have distinct effects on *Sox2* expression. (*A*) Expression fold changes of RT-qPCR data for the *Sox2* gene upon different depletions relative to the geometric mean of the undepleted condition. Statistical significance was assessed using linear mixed-effects models, with *P*-values obtained from a likelihood ratio test comparing models with and without the condition term and adjusted for multiple testing using the Benjamini–Hochberg correction. (*) *P* < 0.05, (**) *P* < 0.01, (***) *P* < 0.001. (*B*) As in *A* but for SCR expression. (*C*) Schematic of the SRR2 deletion experiment. (*D*) Expression fold changes of RT-qPCR data for the *Sox2* gene and the SCR in the SRR2-intact and SRR2-deleted cells with and without CTCF depletion, shown relative to the geometric mean of the undepleted SRR2-intact condition. Individual replicates are shown as points of different shapes, and point colors denote two independent SRR2 deletion clones. Statistical significance assessed using linear mixed-effects models, with *P*-values obtained from a likelihood ratio test comparing models with and without the condition term and adjusted for multiple testing using the Benjamini–Hochberg correction. (*) *P* < 0.05, (**) *P* < 0.01, (***) *P* < 0.001).

To determine whether the loss of 3D genome regulators and the resulting modulation of 3D genome organization affect *Sox2* expression, we performed RT-qPCR for *Sox2* using the degron cell lines. We found a mild significant downregulation of *Sox2* following 3 h of CTCF or RAD21 depletion, which persisted after 6 h of RAD21 depletion but was no longer significant after 6 h of CTCF depletion (Fig. 2A). Thus, a reduction in *Sox2*–SCR looping translates into only modest changes in *Sox2* gene expression, consistent with previous reports showing mild *Sox2* expression changes following loss of cohesin (Aljahani et al. 2022). In contrast, disruption of the *Sox2*–SCR loop through WAPL depletion was associated with a more pronounced significant decrease in *Sox2* expression levels by ∼30% after 6 h (Fig. 2A). Furthermore, we observed that combined depletion of WAPL and CTCF resulted in an even stronger reduction by ∼52% after 6 h (Fig. 2A), suggesting that cohesin stalling at CTCF sites could play a role in maintaining *Sox2* expression when cohesin is stabilized on chromatin. These effects were orthogonally confirmed by reanalyzing previously published RNA-seq data from the respective AID lines (Supplemental Fig. S2A; van Schaik et al. 2022). Together, these results show that different ways of disrupting an enhancer–promoter loop, despite having similar effects on enhancer–promoter contact frequency, can have different effects on gene expression depending on the type of disruption.

One feature of enhancers is the production of unstable enhancer RNA (eRNA), which can be used as a proxy for enhancer activity. To assess whether SCR eRNA production is affected upon RAD21 or WAPL depletion, we made use of the previously published nascent transcription data in the WAPL-AID line (Liu et al. 2021) and generated TT_chem_-seq (Schwalb et al. 2016; Gregersen et al. 2020) data in the RAD21-AID line. Surprisingly, we found that the production of SCR eRNA was mildly increased upon 6 h of RAD21 depletion and more strongly increased upon 6 h of WAPL depletion (Supplemental Fig. S2B). To assay SCR eRNA production in other depletion conditions, we performed RT-qPCR on eRNAs derived from the SCR. Consistent with the nascent transcription data, we detected an increase in SCR transcripts in WAPL-depleted cells and a milder increase in RAD21-depleted cells, showing that we can reliably capture SCR eRNA using RT-qPCR (Fig. 2B). In CTCF-depleted cells, we also observed an increase in SCR transcription, albeit also smaller than upon WAPL depletion (Fig. 2B). To assess whether the eRNA upregulation observed at the *Sox2* locus reflects a more general phenomenon, we analyzed genome-wide intergenic transcripts in the TT_chem_-seq data. Consistent with our locus-specific observation, WAPL depletion resulted in upregulation of intergenic noncoding transcripts more frequently than RAD21 depletion, with 150 and 85 upregulated intergenic transcripts, respectively (Supplemental Fig. S2C). We then asked whether genes located within 200 kb from the intergenic transcripts upregulated upon WAPL depletion also displayed reduced expression, analogous to the observations at the *Sox2* locus. We identified specific examples where gene expression was reduced in WAPL-depleted cells (Supplemental Fig. S2D,E). However, this was not a general trend, as most genes near these transcripts remained unchanged and some were also upregulated (Supplemental Fig. S2D). This is consistent with a previous report suggesting that loss of cohesin loader and extrusion factor NIPBL leads to increased transcription of many noncoding RNAs proximal to enhancers (Schwarzer et al. 2017). Overall, these results indicate that perturbing the 3D genome regulatory proteins results in the overactivation of the SCR, potentially as a compensatory response to the reduction in *Sox2* expression.

### A weak downstream enhancer renders Sox2 expression insensitive to the loss of CTCF

Previous work has assessed the activity of other putative enhancer regions around the *Sox2* gene. *Sox2* regulatory region 2 (SRR2), which is located ∼3 kb downstream from *Sox2*, was found to show enhancer activity in a reporter assay, but deleting this element did not affect *Sox2* expression (Zhou et al. 2014; Hansen et al. 2026). It has recently been shown that deleting SRR2 renders *Sox2* expression sensitive to depletion of cohesin loader and extrusion factor NIPBL, indicating that SRR2 protects *Sox2* against the loss of loop extrusion (Hansen et al. 2026). Considering our results showing that depletion of either RAD21 or CTCF alone does not strongly affect *Sox2* expression, we wondered whether loss of SRR2 might similarly render *Sox2* expression sensitive to the loss of extrusion stalling by CTCF.

To study this, we generated two SRR2 knockout clones in the CTCF-AID degron line using CRISPR–Cas9 genome editing (Fig. 2C). We performed RT-qPCR for *Sox2* in SRR2-intact and SRR2-deleted cells under nondepleted conditions and after 6 and 24 h of CTCF depletion. Consistent with our previous results, *Sox2* expression in cells with intact SRR2 was mildly decreased to ∼73% after 6 h of CTCF depletion and did not decrease further after 24 h (Fig. 2D). Moreover, deletion of SRR2 in undepleted cells resulted in an even milder reduction in *Sox2* expression to 86% of baseline levels (Fig. 2D), consistent with previous work (Zhou et al. 2014; Hansen et al. 2026). In contrast, in SRR2-deleted cells, *Sox2* expression decreased more substantially to ∼59% after 6 h of CTCF depletion and further to ∼54% after 24 h (Fig. 2D). To determine whether these changes were accompanied by altered eRNA production from the SCR, we also performed RT-qPCR for the SCR eRNA. We found that SCR transcription in SRR2-intact cells gradually increased after 24 h of CTCF depletion. However, in SRR2-deleted cells, SCR transcription initially increased after 6 h but dropped to nearly baseline levels after 24 h of CTCF depletion. Together, these results indicate that SRR2 protects *Sox2* expression from the loss of cohesin stalling at CTCF sites and modulates the compensatory response at the SCR.

### BET proteins act additively with the cohesin-mediated loop extrusion to maintain the Sox2–SCR loop

Despite the reduced interaction frequency, there is still residual *Sox2*–SCR looping following acute RAD21 depletion (Fig. 1D), suggesting that there are additional factors beyond loop extrusion involved in establishing and maintaining the *Sox2*–SCR interaction. BET proteins recognize acetylated histones through their bromodomains (Owen et al. 2000; Filippakopoulos et al. 2012), are enriched at superenhancers, and contribute to target gene activation (Cho et al. 2018; Sabari et al. 2018). They are also known to drive long-range chromatin contacts and may be involved in enhancer–promoter communication (Rosencrance et al. 2020). Notably, the SCR is marked by high levels of histone H3 lysine 27 acetylation (H3K27ac) (Supplemental Fig. S3).

**Figure 3.**
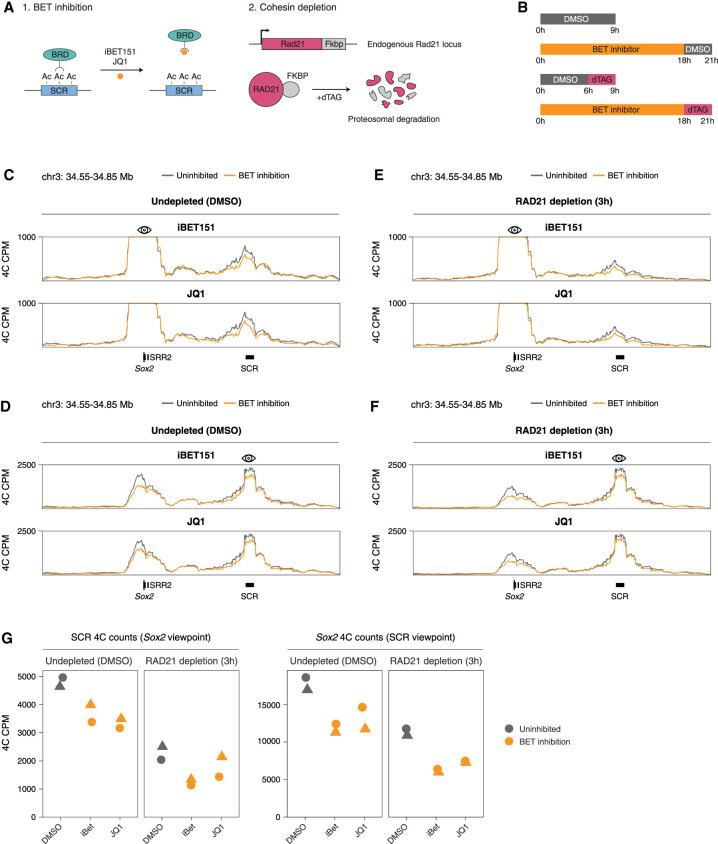
BET proteins act additively with the cohesin-mediated loop extrusion to maintain the *Sox2*–SCR loop. (*A*) Schematic depicting the combined BET inhibition and RAD21 depletion system. (*B*) Schematic depicting the experimental treatment conditions under which the subsequent 4C-seq experiments were performed. (*C*) Normalized 4C-seq coverage of the *Sox2* locus using the *Sox2* promoter viewpoint with and without BET inhibition in the vehicle control (DMSO) condition. (*D*) As in *C* but using the *Sox2* SCR viewpoint. (*E*) Normalized 4C-seq coverage of the *Sox2* locus using the *Sox2* promoter viewpoint with and without BET inhibition in the RAD21-depleted condition. (*F*) As in *E* but using the Sox2 SCR viewpoint. (*G*) Quantification of normalized 4C counts in individual replicates in the SCR using the *Sox2* viewpoint and in the *Sox2* gene using the SCR viewpoint across different conditions.

To understand the interplay between cohesin and BET proteins in the regulation of the *Sox2*–SCR loop, we generated a RAD21 degron cell line using the FKBP system in 129/Castaneus F1 hybrid mESCs (Song et al. 2019) and treated cells with BET inhibitors iBET151 (Dawson et al. 2011) or JQ1 (Fig. 3A; Filippakopoulos et al. 2010). Efficient RAD21 depletion was confirmed by Western blot (Supplemental Fig. S5A). Cells were pretreated with BET inhibitors for 18 h or with DMSO vehicle control for 6 h, followed by 3 h of RAD21 depletion by treatment with dTAG-13 or DMSO (Fig. 3B).

4C experiments in undepleted cells revealed that BET inhibition alone led to a mild but consistent reduction of the *Sox2*–SCR contact frequency compared with DMSO (Fig. 3C,D,G). While previous studies have reported weak or variable effects of BET inhibition on enhancer–promoter looping at other loci (Crump et al. 2021), our data show that BET proteins modestly but reproducibly contribute to *Sox2*–SCR looping (Fig. 3G). On the other hand, RAD21 depletion in cells with uninhibited BET proteins resulted in a more pronounced decrease in contact frequency compared with the DMSO-treated cells (Fig. 3E–G), consistent with our previous results. Importantly, BET inhibitor pretreatment in RAD21-depleted cells also resulted in decreased contact frequency (Fig. 3D–F). The effect of BET inhibition in the absence of cohesin was comparable with that in the undepleted cells. This suggests that binding of BET proteins to SCR and cohesin-mediated loop extrusion contribute to *Sox2*–SCR looping through independent, additive mechanisms.

### Epigenetic silencing of the SCR leads to allelic imbalance in Sox2 expression

**Figure 4.**
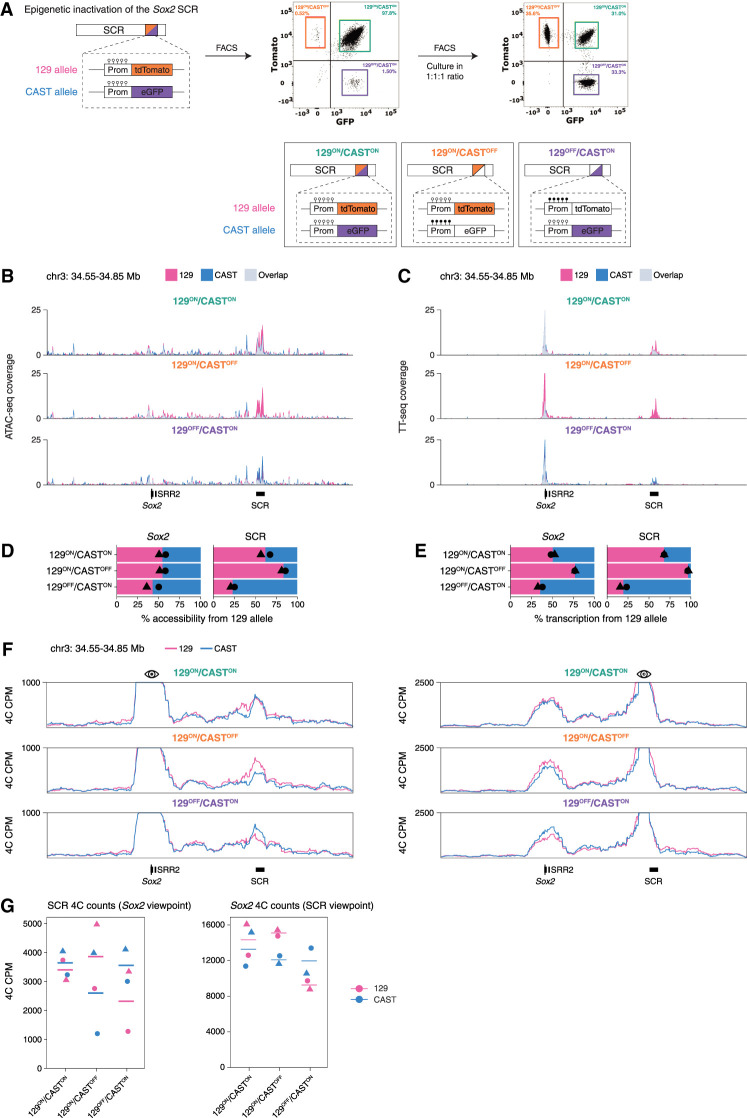
Epigenetic silencing of the SCR leads to allelic imbalance in *Sox2* expression. (*A*) Schematic of a double-sorting protocol to enrich for three populations of the SCR methylation reporter cells in which the SCR is active on both alleles, only 129, or only Castaneus. (*B*) Allele-resolved normalized ATAC-seq coverage tracks in the *Sox2* locus in different methylation reporter populations. (*C*) Allele-resolved normalized TT_chem_-seq coverage tracks in the *Sox2* locus in different methylation reporter populations. (*D*) Quantification of mean allelic proportions of ATAC-seq reads in the 129 allele relative to the sum of the two alleles across different methylation reporter populations. Individual replicates are shown as points of different shapes. (*E*) Quantification of mean allelic proportions of TT_chem_-seq reads in the 129 allele relative to the sum of the two alleles across different methylation reporter populations. Individual replicates are shown as points of different shapes. (*F*) Allele-resolved normalized 4C-seq coverage of the *Sox2* locus across different methylation reporter populations using the *Sox2* promoter viewpoint (*left*) or the SCR viewpoint (*right*). (*G*) Allele-specific quantification of normalized 4C counts in the SCR using the *Sox2* viewpoint and in the Sox2 gene using the SCR viewpoint across different methylation reporter populations. Individual replicates are shown as points of different shapes.

Inhibition of BET proteins may have pleiotropic and indirect effects beyond the inactivation of the SCR. In contrast, DNA methylation can inactivate individual enhancers stochastically in an allele-specific manner, which can be captured using DNA methylation–sensitive reporters (Song et al. 2019). To determine whether SCR activity is specifically required for formation of the *Sox2*–SCR loop, we used a methylation-sensitive fluorescent reporter system previously engineered in the 129/Castaneus F1 hybrid background (Song et al. 2019). In this system, a *Snrpn-tdTomato* fluorescent reporter was inserted into the 129 allele, and a *Snrpn-eGFP* reporter was inserted into the Castaneus allele. These reporters are CpG methylation-sensitive, resulting in their inactivation when methylated. The stochastic epigenetic silencing of the SCR represents an ideal system to determine the contribution of the SCR to enhancer–promoter looping in *cis*. In Figure 4A, we describe a double-fluorescence-activated cell-sorting (FACS) protocol to enrich for three populations of cells in which the SCR is active on (1) both alleles (129^ON^/CAST^ON^), (2) only 129 (129^ON^/CAST^OFF^), or (3) only Castaneus (129^OFF^/CAST^ON^). In unsorted cells, 0.5%–1.5% of cells had an epigenetically silenced SCR (Fig. 4A). We sorted out cells with both alleles active or only one allele active and mixed these populations in a 1:1:1 ratio. After a week of culturing, the three populations remained in nearly a 1:1:1 ratio, suggesting that epigenetic silencing was largely maintained. Targeted amplicon sequencing of bisulfite-treated genomic DNA from the three populations confirmed that the *Snprn* reporter as well as endogenous CpGs in the SCR region adjacent to the reporter were methylated in the inactive alleles and unmethylated in active alleles (Supplemental Fig. S4A).

Next, we wanted to determine whether only the reporter was inactive or whether the entire SCR was inactivated. To this end, we made use of the fact that the 129 and Castaneus alleles have on average a genetic polymorphism every 135 bp. This enabled us to assign short-read sequencing reads to either allele with high confidence (Materials and Methods). Because enhancer activity is often associated with open chromatin, we first performed ATAC-seq in the three populations. In the 129^ON^/CAST^ON^ cells, we found that the SCRs on both alleles are comparably accessible, though we observed the 129 allele to be slightly more accessible. For the 129^ON^/CAST^OFF^ cells, there was a strong bias in the ATAC-seq signal, with ∼84% of reads coming from the 129 allele. For the 129^OFF^/CAST^ON^ cells, this was completely reversed, with only ∼23% of reads coming from the 129 allele. Of note, accessibility of the *Sox2* gene was largely unaffected by the methylation levels and open chromatin state of the SCR (Fig. 4B,D). These results show that the SCR reporter line enables us to select populations in which one of the SCRs is rendered almost completely inaccessible. Interestingly, accessibility of the SCR in the CAST allele in the 129^OFF^/CAST^ON^ cells was increased beyond the levels observed in the 129^ON^/CAST^ON^ cells (Supplemental Fig. S4B). This is consistent with the previous report demonstrating that the active allele can compensate for the loss of expression from the inactive one (Zhou et al. 2014) and suggests that epigenetic inactivation of the SCR on one allele can trigger a response where the remaining SCR on the active allele becomes more accessible.

To measure active transcription, we performed TT_chem_-seq (Schwalb et al. 2016; Gregersen et al. 2020) in the three methylation populations. In the 129^ON^/CAST^ON^ cells, we observed an allelic imbalance in eRNA production at the SCR, with ∼68% of eRNA reads originating from the 129 allele. The proportion of 129 to total reads at the *Sox2* gene was ∼50%, showing that the allelic imbalance in eRNA production in the 129^ON^/CAST^ON^ cells does not translate into an allelic imbalance in *Sox2* gene expression. In the 129^ON^/CAST^OFF^ cells, we found that at the SCR, there were almost no reads coming from the Castaneus allele, with ∼97% of reads originating from the 129 allele. In contrast, in the 129^OFF^/CAST^ON^ cells, the Castaneus allele became the dominant allele, with ∼20% of SCR reads coming from the 129 allele. Importantly, the (long-term) epigenetic inactivation of the SCR on either the 129 or Castaneus allele resulted in reduced *Sox2* expression from the respective alleles (Fig. 4C,E), showing that the inactivation of the SCR is translated into the loss of *Sox2* expression.

Furthermore, in TT_chem_-seq data, we observed a more pronounced compensation effect, as indicated by the increased SCR transcription from the active alleles in the 129^ON^/CAST^OFF^ and 129^OFF^/CAST^ON^ cells beyond the normal levels observed in the 129^ON^/CAST^ON^ cells (Supplemental Fig. S4C). To validate this and obtain absolute quantification of *Sox2* transcripts at the single-cell level, we performed single-molecule fluorescence in situ hybridization (smFISH). To be able to distinguish the 129 from the Castaneus allele, we tagged the 129 allele with MS2v7 loops. We quantified the total levels of *Sox2* with *Sox2* probes and the mRNA counts of the tagged *Sox2* on the 129 allele with MS2 loops in the three populations (Supplemental Fig. S4D). As expected, the total number of *Sox2*–MS2 mRNAs per cell and the fraction of cells with active *Sox2* transcription from the 129 allele were decreased in 129^OFF^/CAST^ON^ cells compared with 129^ON^/CAST^ON^ cells. In contrast, in 129^ON^/CAST^OFF^ cells, both values were increased beyond the levels observed in 129^ON^/CAST^ON^ cells (Supplemental Fig. S4E,F). Moreover, hybridization with the *Sox2* probes indicated that the total number of *Sox2* mRNAs and the fraction of active alleles were comparable in all three populations (Supplemental Fig. S4G,H). Together, these results suggest that epigenetic inactivation of the SCR on one allele can elicit a compensatory response where the SCR on the active allele becomes more accessible and *Sox2* transcript production from the active allele is increased beyond normal levels. Importantly, significance tests for allelic imbalances in the TT_chem_-seq data in 129^ON^/CAST^OFF^ cells compared with 129^OFF^/CAST^ON^ cells revealed that, out of all the transcripts, only the *Sox2* gene and the SCR were significantly affected (Supplemental Fig. S4I). Similarly, in the ATAC-seq data, only SCR accessibility was significantly affected (Supplemental Fig. S4J). These results show that inactivation of the SCR has primarily a local effect. We were unable to detect any chromatin or transcriptional effects beyond the *Sox2* locus, making this an ideal system to assay the contribution of SCR activity to *Sox2*–SCR interactions in *cis*.

### The Sox2–SCR loop is a multicomponent system based on loop extrusion and SCR activity

Next, we aimed to study how the allelic inactivation of a superenhancer affects chromatin looping. To this end, we generated 4C profiles in these cells for the *Sox2* locus. To distinguish the 129 and Castaneus alleles, we performed an allele-specific 4C experiment in which we used a reverse reading primer that amplifies a sequence containing a single nucleotide variant. Following sequencing, ligation product reads from the 129 and Castaneus alleles can be separated computationally (Materials and Methods; Supplemental Fig. S5B). This allows for the detection of both alleles in a single PCR reaction and for measuring the allelic differences with minimal batch effects. In 129^ON^/CAST^ON^ cells, our allele-specific 4C recapitulates the contact profile observed in the degron lines, with a strong interaction between the *Sox2* promoter and the SCR on both alleles (Fig. 4F). In 129^ON^/CAST^OFF^ and 129^OFF^/CAST^ON^ cells, we found that the *cis* interaction frequency between the *Sox2* promoter and the SCR on the inactive alleles is reduced (Fig. 4F,G). Notably, the effect was more pronounced from the *Sox2* promoter viewpoint. This could be explained by the positioning of our allele-specific SCR primers, which lie upstream of the active region of the SCR and may capture interactions that are not strictly reciprocal or as sensitive to SCR inactivation. These results indicate that *Sox2*–SCR looping is in part maintained by SCR activity. However, it is important to note that the overall loop profile remains to a large degree intact despite the fact that the SCR is inactive.

**Figure 5.**
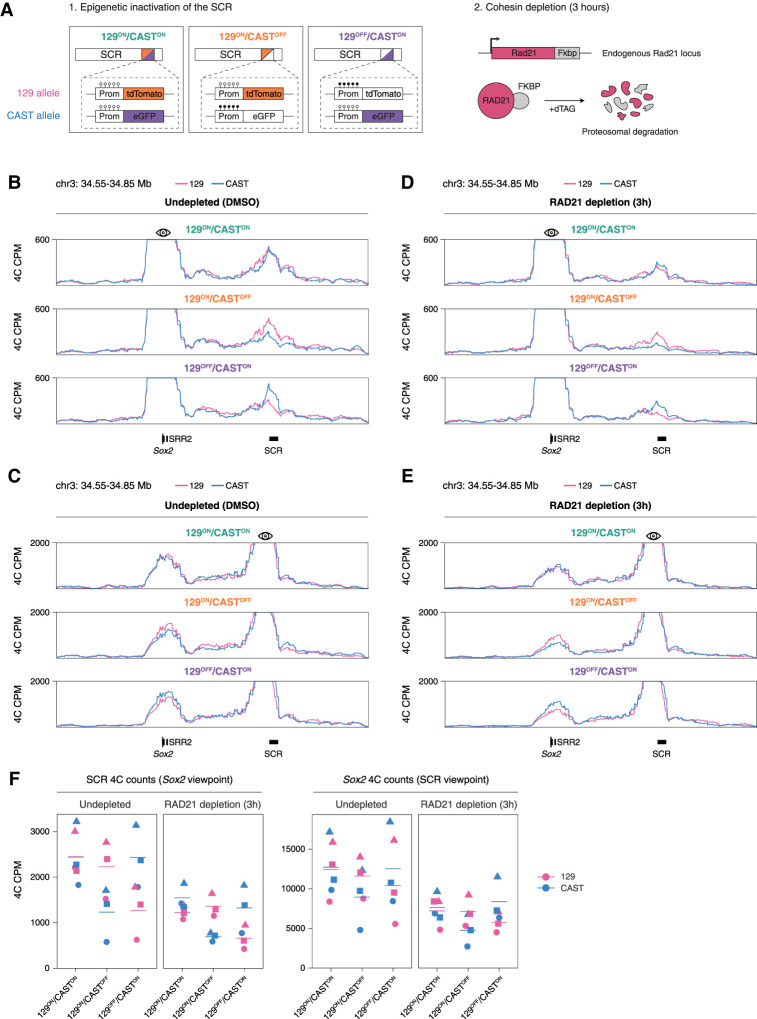
The *Sox2*–SCR loop is a multicomponent system based on loop extrusion and SCR activity. (*A*) Schematic of the RAD21 depletion experiment in the three SCR methylation reporter cell populations. (*B*) Allele-resolved normalized 4C-seq coverage of the *Sox2* locus across different methylation reporter populations in the vehicle control (DMSO) condition using the *Sox2* promoter viewpoint. (*C*) As in *B* but using the SCR viewpoint. (*D*) Allele-resolved normalized 4C-seq coverage of the *Sox2* locus across different methylation reporter populations in the RAD21-depleted condition using the *Sox2* promoter viewpoint. (*E*) As in *D* but using the SCR viewpoint. (*F*) Allele-specific quantification of normalized 4C counts in the SCR using the *Sox2* viewpoint and in the Sox2 gene using the SCR viewpoint across different conditions. Individual replicates are shown as points of different shapes.

To determine the effect of cohesin depletion in the absence or presence of an active or inactive SCR, we made use of the RAD21-FKBP degron line in the allele-specific methylation reporter background, as used in the BET inhibition experiments, and sorted 129^ON^/CAST^ON^, 129^ON^/CAST^OFF^, and 129^OFF^/CAST^ON^ populations (Fig. 5A). We used the double-sorting protocol described above (Fig. 4A) and, following the second sort, let the sorted cells recover for 1 day before depleting RAD21. We performed allele-specific 4C in the three populations treated with DMSO or dTAG-13 to deplete RAD21 for 3 h. In the 129^ON^/CAST^ON^ population, we found that depleting RAD21 resulted in reduced interaction frequency between *Sox2* and the SCR on both alleles (Fig. 5B,C,F), consistent with our previous depletion experiments (Fig. 1D,G). In 129^ON^/CAST^OFF^ and 129^OFF^/CAST^ON^ cells treated with DMSO, we found that *Sox2*–SCR interaction frequency is decreased on the inactive allele (Fig. 5B,C,F), comparable with our previous experiments in cells in which RAD21 was untagged (Fig. 4F,G). When we combined the allelic inactivation with RAD21 depletion, the *Sox2*–SCR interaction frequency on the inactive alleles was further diminished, dropping to nearly background levels observed at the SCR-adjacent regions when using the *Sox2* promoter viewpoint (Fig. 5D–F), indicating a loss of preferential *Sox2*–SCR contacts. Importantly and similarly to our BET inhibition experiments, the effect of SCR inactivation on the Sox2–SCR contact frequency in RAD21-depleted cells was comparable in magnitude with that of the undepleted cells (Fig. 5F). These results show that cohesin-mediated loop extrusion and SCR activity contribute additively to maintaining the *Sox2*–SCR loop. Interestingly, when an SCR viewpoint was used, preferential *cis* contacts in SCR-inactivated alleles persisted even after depleting RAD21 (Fig. 5E,F). This could be due to incomplete methylation of the reporter-adjacent CpG region (Supplemental Fig. S4A, bottom) or due to binding of methylation-insensitive factors that could mediate the 3D organization of this locus.

## Discussion

Regulation of *Sox2* in mouse embryonic stem cells is highly complex, with experiments sometimes yielding paradoxical results (Zhou et al. 2014; Alexander et al. 2019; Aljahani et al. 2022; Taylor et al. 2022). We found that loss of chromatin contacts through depletion of cohesin results in only a mild decrease in *Sox2* expression, which is echoed by recent results for the cohesin loader and extrusion factor NIPBL (Hansen et al. 2026). On the other hand, stabilization of cohesin on chromatin through WAPL depletion and the concurrent loss of contacts between the SCR and the *Sox2* gene do result in a rapid decrease in gene expression. Moreover, we show that loss of cohesin stalling at CTCF sites, while displaying a weak effect on its own, enhances *Sox2* downregulation when combined with WAPL depletion. Additionally, we also found that CTCF is responsible for maintaining *Sox2* expression in the absence of a weak enhancer downstream from *Sox2*. We further show that inactivation of the SCR leads to decreased expression of *Sox2* but only a partial decrease in contact frequency. However, when SCR inactivation is combined with cohesin depletion, contacts are further diminished. Our results demonstrate that the *Sox2*–SCR loop is a multicomponent system dependent on cohesin-mediated loop extrusion, binding of BET proteins, and DNA methylation.

**Figure 6.**
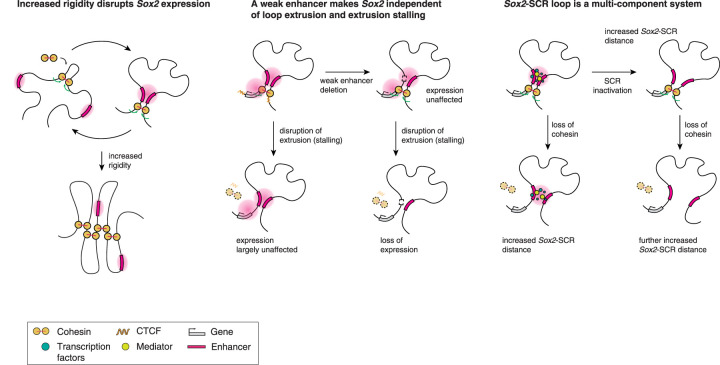
A model of *Sox2*–SCR loop formation and maintenance and the regulation of *Sox2* expression. Increased rigidity of the chromatin by stabilizing cohesin on the DNA lowers the probability of the SCR coming into the proximity of *Sox2* that is necessary for productive transcription initiation. A weak enhancer protects *Sox2* expression against the loss of loop extrusion and extrusion stalling. The *Sox2*–SCR loop is a multicomponent system that additively depends on cohesin-mediated loop extrusion and active enhancer-associated factors.

How do we explain that *Sox2* expression is affected more by the loss of cohesin release factor WAPL than cohesin itself despite the comparable reduction in *Sox2*–SCR contact frequency in our 4C experiments? We hypothesize that loss of cohesin reduces the frequency at which Sox2 and SCR proximity passes the 4C-seq detection threshold (Fudenberg and Imakaev 2017), but through diffusion of the locus or, alternatively, condensate formation, the SCR and *Sox2* can still move into the vicinity, allowing the SCR to activate *Sox2*. On the other hand, loss of WAPL leads to stabilization of cohesin on chromatin, resulting in longer chromatin loops (Liu et al. 2021). Despite the increased cohesin on chromatin, following WAPL depletion, cohesin accumulates at CTCF sites instead of being dynamically reloaded (Liu et al. 2021), leading to decreased chromatin mobility (Mach et al. 2022). This increased rigidity could restrict the diffusion of *Sox2* and the SCR, thereby lowering the probability of the loci coming into the proximity that is necessary for productive transcription initiation (Fig. 6, left).

Furthermore, our data show that *Sox2* expression is more strongly decreased when CTCF is depleted in combination with WAPL. This is not immediately explained by the same model, as the absence of cohesin stalling at CTCF sites should, in principle, make chromatin less rigid (Platania et al. 2024). We propose the following model: When only WAPL is lost, CTCF provides anchoring to the SCR near the *Sox2* promoter, but expression is moderately decreased due to chromatin rigidity. Conversely, when CTCF is lost but WAPL is present, there is no cohesin stalling, but dynamic unloading and loading of cohesin allow it to continuously bring *Sox2* and the SCR into proximity, thus resulting in only a mild reduction in *Sox2* expression. However, when both WAPL and CTCF are lost, cohesin may extrude further without CTCF there to stall it, leading to a loss of SCR anchoring at the *Sox2* promoter, fewer productive Sox2–SCR contacts, and a further decrease in *Sox2* expression. In the future, it would be interesting to explore live-imaging experiments of the *Sox2* locus to quantify and compare the distances and dynamics of the SCR movement relative to the *Sox2* promoter in different depletion settings, as has been done previously for CTCF-mediated loops (Gabriele et al. 2022; Mach et al. 2022; Platania et al. 2024).

There could also be alternative explanations as to how different depletions that we tested resulted in different effects on gene expression despite similar effects on enhancer–promoter contact frequency. For example, it has been reported previously that cohesin, but not CTCF, affects search time of transcription factors (TFs) (Hsieh et al. 2022; Rinaldi et al. 2022), including SOX2 and OCT4 (Hsieh et al. 2022) that also bind to the SCR (Zhou et al. 2014), which could also play a role in our system. It remains to be explored how stabilizing cohesin on chromatin by depleting WAPL affects general TF occupancy and search time.

One clue into how *Sox2* expression is maintained in the absence of loop extrusion lies in the role of the weak downstream enhancer SRR2. SRR2 was previously shown to sustain *Sox2* transcription in the absence of NIPBL, which results in a massive decrease in loop extrusion (Hansen et al. 2026). We show here that SRR2 is also crucial to maintain expression in the absence of CTCF, suggesting that the stalling of cohesin, likely near the promoter, is crucial for maintaining *Sox2* transcription (Fig. 6, middle). This is somewhat surprising, as the contact frequency between Sox2 and the SCR was hardly affected following CTCF depletion, indicating that even when we detect interactions between promoters and enhancers, we must be cautious in interpreting these as functional in terms of promoting gene expression. One way that SRR2 could regulate SCR–*Sox2* communication is through specific classes of regulatory elements, such as facilitators (Blayney et al. 2023) and range extender elements (Bower et al. 2025), which have little activity on their own but do play a synergistic role with enhancers in driving gene expression. A well-studied example of this is LDB1, a transcription factor involved in chromatin looping in erythroid cells (Deng et al. 2012; Krivega et al. 2014; Aboreden et al. 2025). However, *Sox2* expression is unaffected by the knockout of LDB1 (Hansen et al. 2026), indicating that this mechanism cannot explain the enhancer synergy in the *Sox2* locus. Furthermore, SRR2 deletion had no effect on the interaction profiles in the *Sox2* locus as measured by 4C (Hansen et al. 2026). How SRR2 functions to relay information from the SCR to the *Sox2* promoter is an intriguing avenue to investigate in the future.

Previous work has shown that *Sox2* expression levels are controlled by a negative feedback loop (Ormsbee Golden et al. 2013), which is confirmed by our own findings that *Sox2* transcription increases following acute depletion of SOX2 protein (Maresca et al. 2023). Here we report that SCR activity is upregulated in response to reduced *Sox2* transcription. One possibility is that this occurs as a direct consequence of decreased SOX2 levels, with the SCR responding to lower levels of SOX2. This model is compatible with the short half-lives of both the SOX2 transcript (∼1–2 h) (Sharova et al. 2009) and protein (∼5–6 h) (Fang et al. 2014), which would allow the enhancer to rapidly respond to changes in SOX2 transcript or protein abundance. Alternatively, the reduction of cohesin at the SCR and the decrease in enhancer–promoter contacts may directly affect eRNA production at the SCR. An intriguing possibility is that, within the *Sox2*–SCR regulatory hub, RNA polymerase II is loaded at the SCR and transferred to the *Sox2* promoter. Disruption of the *Sox2*–SCR loop could prevent this transfer, leading to the accumulation of RNA polymerase II and increased transcription at the SCR. This form of cohesin–transcription antagonism through competition with a promoter is consistent with a previous observation that loss of NIPBL leads to increased noncoding transcription proximal to enhancers (Schwarzer et al. 2017). However, whether the increased SCR activity is required to maintain *Sox2* transcription in the absence of loop extrusion and extrusion stalling remains an interesting question for future investigation.

The *Sox2*–SCR loop is revealed here to be a multicomponent system comprising loop extrusion and enhancer activity, where both targeted SCR methylation and pleiotropic inhibition of BET proteins resulted in a decrease of *Sox2*–SCR contacts (Fig. 6, right). This raises the question of which upstream factors could be involved in recruiting BET proteins to enhancers and which downstream factors mediate enhancer–promoter contacts. It has been shown that TFs can recruit BET protein BRD4 to its binding sites through either p300-mediated acetylation of histones or acetylation of TFs that can directly interact with BRD4 (Roe et al. 2015). Future research is necessary to systematically dissect which TFs are responsible for mediating BET protein recruitment and chromatin interactions at the SCR.

Enhancer clusters, like the SCR, are characterized by high levels of Mediator binding, making Mediator a possible candidate in mediating enhancer–promoter contacts. Early ChIP-seq experiments reported substantial overlap between Mediator subunits and NIPBL, leading to a model in which the Mediator complex is instructive for loop formation (Kagey et al. 2010; Lai et al. 2013). However, subsequent work employing acute depletions of Mediator subunits was unable to detect changes in the Hi-C and H3K27ac Hi-ChIP maps (El Khattabi et al. 2019; Jaeger et al. 2020). More recently, high-resolution microcapture-C (MCC) experiments did reveal a role for the Mediator complex in the formation of long-range chromatin contacts and the binding of the cohesin subunit SMC1A to chromatin (Ramasamy et al. 2023). However, the interplay between chromatin looping and Mediator function is difficult to tease apart because of the direct role that Mediator plays in transcription regulation. Additionally, superresolution microscopy has revealed that Mediator and BRD4 can form nuclear condensates (Cho et al. 2018; Zamudio et al. 2019). It is tempting to speculate that these condensates are involved in the sequestration of genomic loci and promote chromatin looping, which would be consistent with our BET inhibition data, though the evidence for this is still limited. Other mechanisms, including direct protein–protein interactions, may also potentially drive this form of interaction; for examples, see Magnitov and de Wit (2024).

Our results also show that inactivation of the SCR on a single allele affects only the SCR and *Sox2* transcription on the same allele. Enhancer clusters have been previously shown to often engage in multiway interactions across large distances (de Wit et al. 2013; Beagrie et al. 2017), but whether these interactions had functional consequences for the long-distance interactions has been the subject of debate (Noordermeer et al. 2011). Our observation that there are no detectable changes in any of the genes interacting on the same chromosome—or any other genes—suggests that the SCR activity is not important for the activity of other enhancer clusters and the genes regulated by them and that the regulatory effects of the SCR in mESCs are limited to the *Sox2* locus.

Our work focuses specifically on the *Sox2* locus, bringing into question how these results apply genome-wide. While we believe that the components that we identified (namely, loop extrusion and enhancer-associated factors) drive loop formation at many loci, the *Sox2* locus contains unique features. The SCR is one of the few regions that still form an insulation boundary even after CTCF depletion (Nora et al. 2017; Taylor et al. 2022). This may be due to the SCR being a preferred cohesin loading site, explaining the remaining boundary following CTCF depletion. In the future, it will be interesting to identify and explore whether other loci that show stronger dependency on WAPL than RAD21 behave similarly to the *Sox2* locus.

In summary, our analyses reveal an intricate interplay between enhancer activity and loop extrusion in the establishment and maintenance of chromatin loops. To understand how 3D genome contacts and the underlying protein complex collaborate to achieve gene activation, careful dissection of individual loci is still required. We envisage that a better understanding of the underlying mechanisms will enable us to develop more predictive models of how expression of developmentally important genes is activated.

## Materials and methods

### Cell culture

Mouse embryonic stem cell lines were cultured as described previously (Liu et al. 2021, 2025; van Schaik et al. 2022). Briefly, the degron-tagged cell lines and the 129/Castaneus F1 hybrid methylation reporter cell lines were cultured on 0.1% gelatin-coated plates in serum-free DMEM/F12 (Gibco) and neurobasal (Gibco) medium (1:1) supplemented with N-2 (Gibco), B-27 (Gibco), 0.05% BSA (Gibco), 10^4^ U of leukemia inhibitory factor (LIF; Millipore), 1 µM MEK inhibitor PD0325901 (Selleckchem), 3 µM GSK3-β inhibitor CHIR99021 (Cayman Chemical), and 1.5 × 10^−4^ M 1-thioglycerol (Sigma-Aldrich). The cell lines were passaged every 2 days in daily culture.

### Establishing a RAD21-FKBP degron line

For targeting of the endogenous RAD21 protein, a previously published Rad21 donor plasmid was used (Liu et al. 2025). The neon transfection system (Thermo Fisher) was used to electroporate the donor plasmid and the corresponding sgRNA (pX330-EN1082, Addgene 156 450) into F1 hybrid methylation reporter cell lines (Song et al. 2019). BFP-positive cells were sorted into a gelatinized 96 well plate for single-clone selection. PCR was used to genotype the clones, and the FKBP fusion was validated by Sanger sequencing. RAD21 depletion after 3 and 4 h of dTAG-13 treatment was confirmed by Western blot.

### Acute protein degradation and inhibition of BET proteins

CTCF, RAD21, and WAPL proteins were depleted by treating the AID-tagged cell lines with a final concentration of 500 µM IAA (Sigma-Aldrich) and harvesting the samples at different time points after treatment. In RAD21-FKBP lines, RAD21 was depleted by adding a final concentration of 500 nM dTAG-13 molecule (Merck Millipore). For BET inhibition experiments, the RAD21-FKBP lines were first pretreated with either DMSO (vehicle control) for 6 h or small molecule inhibitors iBET151 or JQ1 (Tocris) at a concentration of 1 µM for 18 h prior to 3 h of dTAG-13 treatment.

### Sorting of the methyl reporter populations

The isolation of the different methyl reporter populations by FACS was performed based on reporter fluorescence: tdTomato^+^/eGFP^+^ (129^ON^/CAST^ON^), tdTomato^+^/eGFP^−^ (129^ON^/CAST^OFF^), and tdTomato^−^/eGFP^+^ (129^OFF^/CAST^ON^). For the first enrichment step, ∼100 million cells were used as input. Sorted cells were immediately replated in fresh medium without centrifugation or washing. After 5–6 days, a second FACS enrichment was performed, yielding sufficient cell numbers of the three populations for subsequent experiments. Where indicated, cells were treated with DMSO/dTAG-13 for 3 h prior to subsequent sample collection.

### 4C-seq

High-resolution 4C-seq was performed as described previously with a two-step PCR method for indexing (van de Werken et al. 2012; Haarhuis et al. 2017) on the AID- and FKBP-tagged cell lines and the sorted methylation reporter populations. Ten million cells were used for each sample. DpnII and Csp6I (NEB) were used as the primary and secondary restriction enzymes, respectively. For allele-resolved 4C-seq, a reverse reading primer that amplifies a single nucleotide variant was used. An overview of the viewpoint primers used is provided in Supplemental Table S1. Paired-end sequencing of the libraries was carried out on an Illumina NextSeq 550. 4C-seq on the sorted methylation reporter populations was done in triplicate, and other 4C-seq experiments were done in duplicate.

### RT-qPCR

RNA was isolated from the AID-tagged and SRR2-deleted cell lines using RNeasy kit (Qiagen 74106) and treated with DNase I. cDNA was synthesized using the iScript cDNA synthesis kit (Bio-Rad). Expression of the *Sox2* gene and the *Sox2* SCR was quantified by RT-qPCR using a SensiFAST No-ROX kit (Bioline) with primers for the housekeeping *Rps26* gene, *Sox2*, and the SCR. The primers used are listed in Supplemental Table S2. RT-qPCR was performed in quadruplicate and across two individual clones for the SRR2-deleted cells. The *Ct* values for the *Sox2* gene and the SCR were normalized to the *Rps26* values to obtain Δ*Ct* values. Expression ratios (2^–ΔΔ*Ct*^) were computed relative to the geometric mean of the reference conditions. Due to observed systematic differences between replicates, differential testing between conditions was performed on Δ*Ct* values using a linear mixed model with a replicate as a random intercept and implemented in the lme4 package (Bates et al. 2015).

### ChIP-qPCR

About 20 million embryonic stem cells were cross-linked for 10 min with 1% formaldehyde and quenched with 2.0 M glycine. The cross-linked cells were lysed and sonicated on a Bioruptor Pico (Diagenode) for five cycles of 30 min on and 30 min off. Anti-Rad21 antibody (5 µg/chip; Abcam ab154769) was coupled to protein G beads and incubated overnight with the sonicated lysate. After washing, elution, and decross-linking, the captured chromatin template was purified with the PCR purification kit (Qiagen 28106). ChIP templates were mixed with SensiFAST SYBR No-ROX mix (GC Biotech B.V. BIO-98005) and the primers listed in Supplemental Table S2. qPCR was performed on QuantStudio5 (Thermo Fisher). The experiment was performed in duplicate.

### ATAC-seq

ATAC-seq on sorted methylation reporter populations was carried out following a previously described protocol (Buenrostro et al. 2013). Cells were sorted based on their fluorescence signal into independent tubes, and 50,000 cells were used for ATAC. The Tn5 transposase was purified in-house by the Netherlands Cancer Institute Protein Facility as described by Braccioli et al. (2025). Cells were collected and washed once in cold PBS, and nuclei were lysed in 2× lysis buffer (1 M Tris-HCl at pH 7.5, 5 M NaCl, 1 M MgCl_2_, 10% Igepal). The nucleus isolation buffer was removed by centrifugation, and nuclei were resuspended in tagmentation buffer [20 mM Tris (hydroxymethyl)aminomethane, 10 mM MgCl_2_, 20% dimethylformamide adjusted to pH 7.6 with acetic acid] containing Tn5 and incubated for 1 h at 37°C. Two rounds of PCR were performed using the KAPA kit to incorporate sequencing adapters and to amplify the library DNA. Libraries were size-selected with AMPure XP beads (Beckman) to retain fragments <700 bp. ATAC-seq experiments were performed in duplicate.

### TT_chem_-seq

TT_chem_-seq on sorted methylation reporter populations and in the RAD21-AID line was performed following an established protocol (Gregersen et al. 2020). Briefly, cells were labeled for 10 min using 2 mM 4SU, total RNA was isolated and fragmented, and streptavidin-coated MicroBeads were used to enrich the 4SU-biotin-labeled RNA. KAPA RNA HyperPrep kit (Roche) with dual indexing adapters was used to prepare the libraries, which were sequenced on a NextSeq 550. TT-seq experiments were performed in duplicates.

### Bisulfite conversion and amplicon sequencing

Genomic DNA was extracted from each sorted population (200,000 cells per condition), which served as input for bisulfite conversion, which was carried out using the EpiJET bisulfite conversion kit (Thermo K1461) following the manufacturer's instructions. The reporter region was first amplified using allele-specific primers, followed by a second (nested) PCR utilizing internal primer pairs. Amplified products were gel-purified and submitted for Sanger sequencing. For each CpG site, the ratio of reads containing a cytosine instead of thymine was used to estimate the percentage of methylation. In addition to the reporter region itself, the adjacent SCR region was also amplified and sequenced using the MiSeq Nano system. An overview of the primers used for the reporter and adjacent SCR region is provided in Supplemental Table S3. Sequencing reads were aligned using Bismark (v0.22.3) (Krueger and Andrews 2011). Two single-nucleotide polymorphisms within the amplicon were used to assign aligned reads into CAST- or 129-specific reads, and Bismark was used to perform methylation calling on allele-specific reads.

### Establishing a Sox2–MS2v7 fusion gene

The MS2v7 repeats (Vera et al. 2019) were introduced into the 129 allele of the *Sox2* 3′ UTR in cells containing DNA methylation reporter in the SCR (Song et al. 2019). Cells were electroporated with 2 µg of a plasmid containing Cas9 and the scaffold encoding an allele-specific gRNA at the *Sox2* 3′ UTR, as well as 2 µg of a targeting vector containing 24 copies of the MS2v7 repeats, a HaloTag flanked by loxP sites, and primer binding sites, which were used to facilitate screening for inserted clones, following the strategy described by Brouwer et al. (2024). After labeling with JF646, transfected cells were selected for fluorescence. Individual positive cells were sorted into 96 well plates for clonal amplification and screened for heterozygous allele-specific incorporation of full-length 24xMS2 repeats by PCR and sequencing.

### Single-molecule RNA fluorescent in situ hybridization (smFISH)

After sorting of eGFP^+^, tdTomato^+^, or double-positive cells, 400,000 cells were seeded onto poly-L-lysine-coated coverslips in culture medium and incubated for 2–3 h at 37°C and 5% CO_2_ to attach to the coverslips. Cells were washed three times with warm Hank's buffered salt solution (Gibco), fixed with 4% paraformaldehyde for 10 min, and washed twice for 10 min in PBS. The cells were permeabilized with 70% ethanol overnight at 4°C. Coverslips were hybridized overnight at 37°C with hybridization buffer containing 10% dextran sulfate, 10% formamide, 2× SSC, and 5 pmol of fluorescent probes targeting *Sox2* or *MS2v7* and labeled with Quasar 670 dye (Biosearch Technologies). Coverslips were washed three times for 30 min with 10% formamide and 2× SSC at 37°C, once with 2× SSC, and once for 5 min with PBS at room temperature. Coverslips were mounted on microscope slides using ProLong Gold mounting medium containing DAPI (Thermo Fisher). Coverslips were dried for at least 24 h at room temperature in the dark before imaging. Imaging was performed on an inverted microscope (Zeiss AxioObserver) with a plan-apochromat 40× 1.4 NA oil DIC UV objective, a 1.60× optovar, and an sCMOS camera (Hamamatsu Orca Flash 4v3). For Quasar 670, a 660 nm longpass dichroic (Chroma T660lpxrxt), a 697/60 nm emission filter (Chroma ET697/60 m), and 640/30 nm LED excitation at full power (Spectra X, Lumencor) were used. For DAPI, a 425 nm longpass dichroic (Chroma T425lpxr), a 460/50 nm emission filter (Chroma ET460/50 m), and LED excitation at 395/25 nm at 25% power (Spectra X, Lumencor) were used. For each sample and each channel, we used Micro-Manager to acquire several fields of view, each consisting of 27 *Z*-stacks (Δ*z* 0.3 µm) at 25 msec exposure for DAPI and 750 msec exposure for Quasar 670.

### SRR2 deletion

Endogenous deletion of the SRR2 element in the CTCF-AID cell line was performed using previously described gRNAs (sequences are in Supplemental Table S4; Hansen et al. 2026) cloned into the PX458-GFP vector [pSpCas9(BB)-2A-GFP, Addgene plasmid 48138]. The gRNA constructs were transfected into CTCF-AID cells with the FuGENE HD transfection reagent (Promega). Forty-eight hours after transfection, GFP-positive cells were sorted by flow cytometry and grown for 5 days. Subsequently, GFP-negative cells were single-sorted into a 96 well plate for single-clone selection. PCR was used to genotype the clones, and the SRR2 deletion was validated by Sanger sequencing. Two clones were used for the RT-qPCR analysis.

### 4C-seq analysis

To create the restriction fragment maps with DpnII and Csp6I digestion, R/Bioconductor package Biostrings (https://www.bioconductor.org/packages/Biostrings) was used to scan the mm10 mouse reference genome for restriction enzyme recognition sites. The fragments were filtered for those that contained both the primary (DpnII) and secondary (Csp6I) restriction sites. Viewpoints were demultiplexed by scanning raw reads for viewpoint-specific primer sequences using Biostrings while allowing for up to two mismatches and trimming the reads up to the primary restriction site. For the allele-resolved 4C, the reads were further split into alleles based on the sequenced bases at the SNP positions located downstream from the reverse primers (listed in Supplemental Table S1). The trimmed reads were aligned to the mm10 genome using bwa-aln (v0.7.17-r1188) (Li and Durbin 2009) and filtered for paired and uniquely mapped reads using SAMtools (v1.19.2) (Li et al. 2009). Fragment counts were generated by counting the reads with exact matches of start or end positions to fragment boundaries. The fragment counts were scaled to the total library counts per million and smoothed using a rolling mean with a window size of 21 fragments.

### Assigning sequencing reads to alleles

To resolve allele-specific reads, ATAC-seq and TT_chem_-seq data were mapped separately to CAST/EiJ and 129S1/SvImJ genome assemblies (Keane et al. 2011). For each read, the alignment score (AS) tag from the BAM files was compared between alleles. Reads were assigned to the allele with the higher AS value, while reads with equal AS for both alleles were deemed unassigned. Subsequently, the reads were lifted over to the mm10 reference genome for quantification based on reference coordinates.

### ATAC-seq data processing

ATAC-seq data were mapped to the mm10 genome using bwa-mem (v0.7.17-r1188) (Li and Durbin 2009) and filtered for paired and uniquely mapped reads using SAMtools (v1.19.2) (Li et al. 2009). The reads were assigned to 129 or CAST alleles as described. Coverage peaks were called using macs2 (Zhang et al. 2008). Read counts in peaks were generated using the “summarizeOverlaps” function from the R/Bioconductor package GenomicAlignments (Lawrence et al. 2013) with the mode “Union.”

### TT_chem_-seq data processing

TT-seq data were mapped to the mm10 genome using the STAR aligner (v2.7.9) (Dobin et al. 2013) and filtered for paired and uniquely mapped reads using SAMtools (v1.19.2) (Li et al. 2009). The reads were assigned to 129 or CAST alleles as described. Active transcription units were called by fitting a two-state HMM model using GenoSTAN (Zacher et al. 2017). Read counts in transcription units were generated using the “summarizeOverlaps” function from the R/Bioconductor package GenomicAlignments (Lawrence et al. 2013) with the mode “Union.”

### Allelic imbalance quantification

ATAC-seq and TT_chem_-seq read counts were normalized using DESeq2 (Love et al. 2014) with library size set to the total number of unassigned reads. The allelic proportion of *Sox2* and SCR reads was calculated by dividing the normalized read count for each allele by the sum of the counts from both alleles. For each sample, the difference in 129/CAST allelic ratios between 129^ON^/CAST^OFF^ and 129^OFF^/CAST^ON^ conditions was quantified by fitting a model with an interaction term between allele and condition and testing significance using the Wald test in DESeq2.

### smFISH analysis

A custom Python script was used to detect, localize, and classify the spots (https://www.github.com/Lenstralab/smFISH). Cells and nuclei were segmented using Otsu thresholding and watershedding. Spots were localized by fitting a 3D Gaussian mask after local background subtraction (Coulon et al. 2014) and counted per cell. Cells in which no spots were detected were excluded from further analysisbecause a visual inspection indicated that these cells were not properly segmented. The fraction of active alleles was determined by normalizing the brightest (for MS2 probes) or two brightest (for *Sox2* probes) nuclear spots to the median fluorescent intensity of the cytoplasmic RNAs detected in all cells. Nuclear foci containing ≥2.5 RNAs were classified as active transcription sites.

### Data analysis and visualization

Statistical analyses were performed in R (v4.3.3; https://www.R-project.org) using Bioconductor (v3.18) (Gentleman et al. 2004) packages where indicated. Visualizations were generated using the ggplot2 package (https://ggplot2.tidyverse.org).

### Data availability

All sequencing data generated as part of this study have been deposited to Gene Expression Omnibus (GEO) under accession number GSE306301.

## Supplemental Material

Supplement 1

Supplement 2

Supplement 3

Supplement 4

Supplement 5
