# Supplementary material for: Cohesin-mediated loop extrusion and enhancer-associated factors additively contribute to Sox2 looping with its distal enhancer
Source: Genes Dev. 2026 Jun 1;40(11-12):893–911. doi: 10.1101/gad.353296.125 (PMC13224857; doi:10.1101/gad.353296.125)
Supplement: Supplement 1 [file Supplemental_Figures.pdf]

Supplemental Figure S1

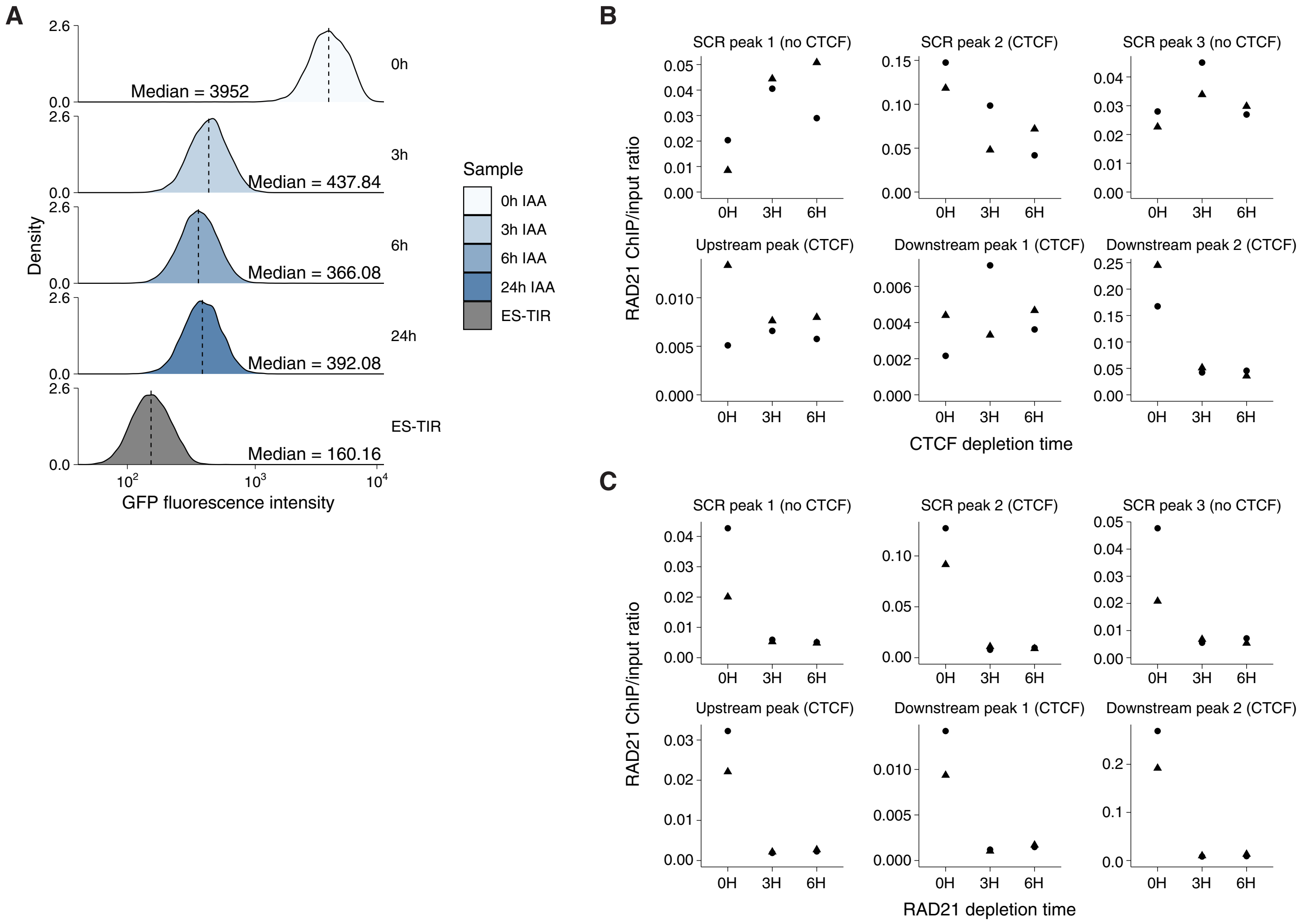

Supplemental Figure S1. Validation of CTCF degradation in the CTCF-AID line.

(A) GFP fluorescence intensity across the CTCF depletion time course and control cell line with untagged CTCF.

(B) RAD21 ChIP-qPCR fold changes relative to input at six RAD21 peaks in the *Sox2* locus upon 3 or 6 hours of CTCF depletion. Different point shapes denote the two replicates.

(C) RAD21 ChIP-qPCR fold changes relative to input at six RAD21 peaks in the *Sox2* locus upon 3 or 6 hours of RAD21 depletion. Different point shapes denote the two replicates.

Supplemental Figure S2

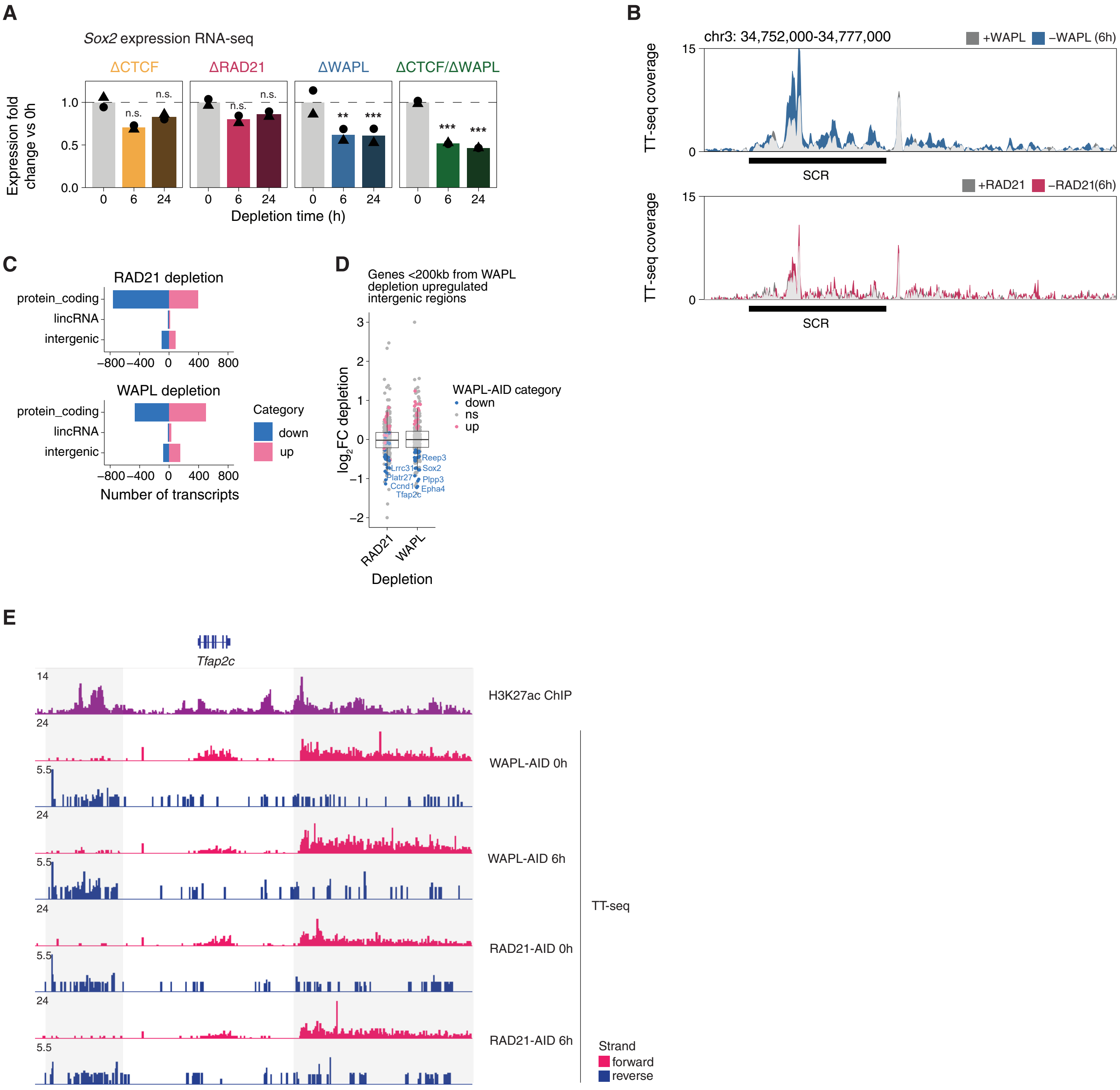

Supplemental Figure S2. WAPL depletion leads to increased eRNA production at the SCR.

(A) Expression fold changes of RNA-seq data for the *Sox2* gene upon different depletions relative to the mean of the undepleted condition. Statistical significance was determined using the Wald test in DESeq2 (*p<0.05 **p<0.01 ***p<0.001). Data from van Schaik et al., 2022. Individual replicates are shown as points of different shapes.

(B) TT_{chem}-seq coverage in the *Sox2* locus and the zoom-in on the SCR with and without WAPL/RAD21 depletion. WAPL-AID data from Liu et al. 2021.

(C) Number of identified differentially expressed transcripts in WAPL/RAD21 depletion TT_{chem}-seq data. Significant transcripts were defined as padj < 0.05 using DESeq2.

(D) Log fold changes in WAPL/RAD21 depletions of genes with transcription start sites located less than 200 kb from WAPL depletion-upregulated intergenic transcripts.

(E) Example locus showing the downregulated *Tfap2c* gene with two non-coding transcripts identified as upregulated in WAPL depletion (shaded boxes).

Supplemental Figure S3

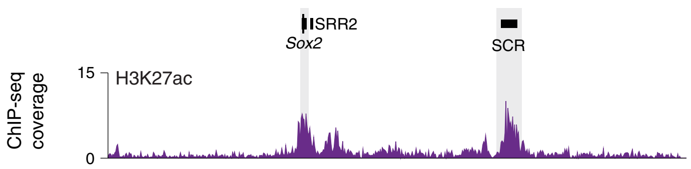

Supplemental Figure S3. SCR displays high levels of histone H3 lysine 27 acetylation.

H3K27ac ChIP-seq coverage in the *Sox2* locus. Data from Liu et al. 2021.

Supplemental Figure S4

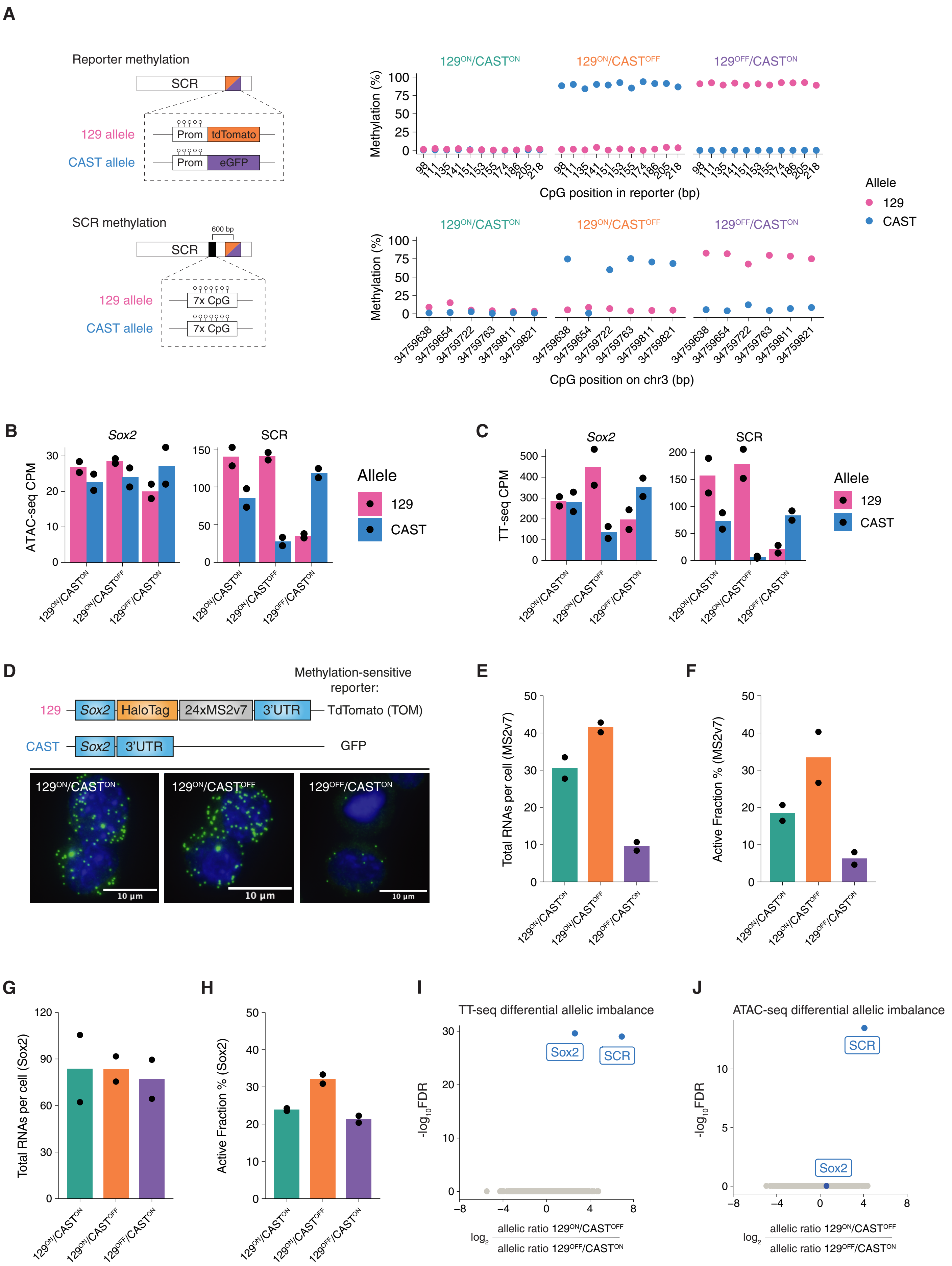

Supplemental Figure S4. Epigenetic inactivation of the SCR on one allele can trigger a compensatory response on the other allele.

(A) Proportions of methylated CpGs in the methylation reporter (top) and in the adjacent SCR region (bottom) across different methylation reporter populations

(B) ATAC-seq read counts per million in the *Sox2* gene and the SCR in the two alleles across different methylation reporter populations.

(C) TT_{chem}-seq read counts per million in the *Sox2* gene and the SCR in the two alleles across different methylation reporter populations.

(D) Schematic (top) and the representative images (bottom) of the smFISH experiment to visualize *Sox2* transcripts from the MS2v7 loop-tagged 129 allele.

(E) smFISH quantification of the MS2v7-tagged *Sox2* RNAs per cell from the 129 allele in different methylation reporter populations.

(F) smFISH quantification of the proportion of cells with active MS2v7-tagged *Sox2* transcription on the 129 allele in different methylation reporter populations.

(G) smFISH quantification of the total *Sox2* RNAs per cell in different methylation reporter populations.

(H) smFISH quantification of the proportion of cells with active *Sox2* transcription in different methylation reporter populations.

(I) Differential allelic imbalance testing in the TT_{chem}-seq data. The x axis represents the log fold change in the 129/CAST read count ratios in the $129^{ON}/CAST^{OFF}$ population compared to the $129^{OFF}/CAST^{ON}$ population of cells.

(J) Differential allelic imbalance testing in the ATAC-seq data. The x axis represents the log fold change in the 129/CAST read count ratios in the $129^{ON}/CAST^{OFF}$ population compared to the $129^{OFF}/CAST^{ON}$ population of cells.

Supplemental Figure S5

A

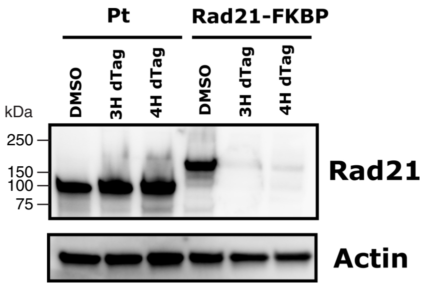

B

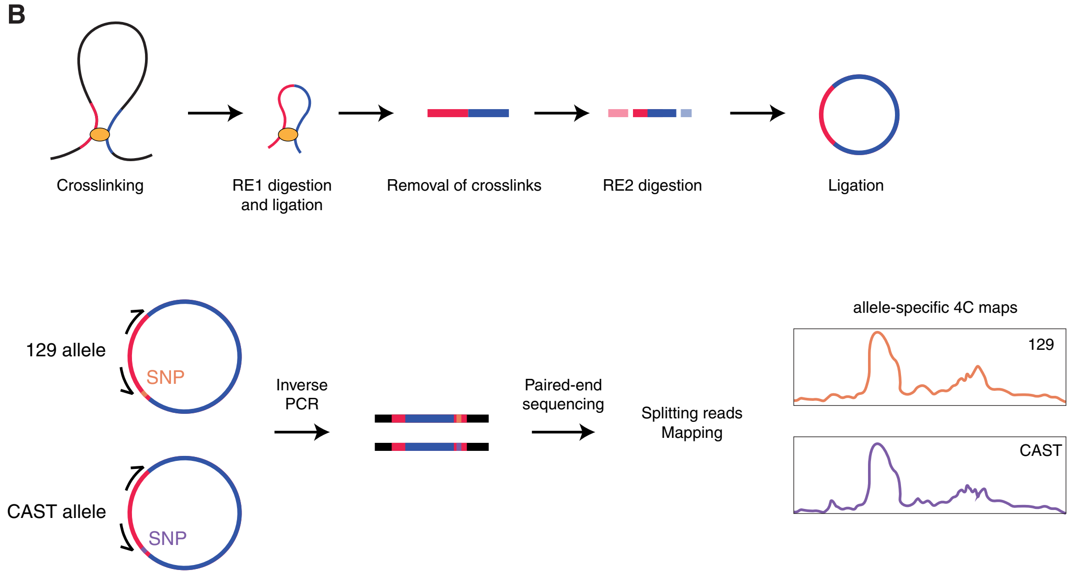

Supplemental Figure S5. Cohesin depletion and generation of allele-resolved 4C profiles in RAD21-FKBP 129/Castaneus F1 hybrid cell line.

(A) Western blot for RAD21 in parental and in RAD21-FKBP 129/Castaneus F1 hybrid mESCs, treated with DMSO vehicle or dTAG-13 to deplete RAD21.

(B) Schematic of the allele-specific 4C experiment and the quantification of allele-specific reads.
